# Impact of implementing performance-based financing on childhood malnutrition in Rwanda

**DOI:** 10.1186/1471-2458-14-1132

**Published:** 2014-11-04

**Authors:** Agnes Binagwaho, Jeanine Condo, Claire Wagner, Fidele Ngabo, Corine Karema, Steve Kanters, Jamie I Forrest, Jean de Dieu Bizimana

**Affiliations:** Ministry of Health, Government of Rwanda, Kigali, Rwanda; Department of Global Health and Social Medicine, Harvard Medical School, Boston, USA; University of Rwanda, College of Medicine and Health Sciences, School of Public Health, Kigali, Rwanda; Global Health Delivery Partnership, Boston, USA; Malaria & Other Parasitic Diseases Division- RBC, Kigali, Rwanda; School of Population and Public Health, University of British Columbia, Vancouver, Canada; World Bank, Washington, DC USA; Geisel School of Medicine, Dartmouth College, Hanover, USA

**Keywords:** Malnutrition, Maternal health, Child health, HIV/AIDS, Performance-based financing

## Abstract

**Background:**

Malnutrition remains a serious concern in Rwanda, particularly among children under-5 years. Performance-based financing (PBF), an innovative health systems financing strategy, has been implemented at the national level since 2008. This study aimed to assess the impact of PBF and other factors associated with the prevalence of three classifications of malnutrition (stunting, wasting and underweight) in children under-5 years in Rwanda.

**Methods:**

The study is a cross-sectional study comprising of 713 children under five years old from 557 households, whose anthropometric measurements (height, weight and age) had been obtained as part of the 2008 Rwanda General Health and HIV household survey. Z-scores for height-for-age, weight-for-age, weight-for-height, and body mass index-for-age were analyzed according to the World Health Organization 2006 Child Growth Standards. Random intercept logistic regression models were used to regress each anthropometric measure (WAZ, HAZ and WHZ) against child, maternal and household characteristics.

**Results:**

Child participants ranged in age from 0 to 60 months, 20.2% of children were under 12 months and 5.1% were HIV positive. The prevalence of wasting was 8.8%; of stunting was 58.4%; and of underweight status was 20.7%. Maternal emotional and social wellbeing was protective of wasting in children under-5 years of age. Living in districts implementing PBF was protective of wasting (Adjusted Odds Ratio: 0.43; 95% confidence interval: 0.19-0.97). Living in a district with PBF was not found to be associated with either stunting or underweight status among children under-5.

**Conclusions:**

PBF may have a protective association with particular forms of malnutrition among children under-5 years in Rwanda. These findings warrant further investigation in relation to the impact of implementing innovative financing schemes on health outcomes.

## Background

Malnutrition remains a serious concern in Rwanda, like many regions in Africa. The continent is home to more than 31 million underweight children who disproportionately suffer from infection due to depressed immune function; indeed malnutrition accounts for more than one-third of post-natal deaths [1]. In Rwanda, under-nutrition can be attributed to about half of all pediatric deaths [2]. Although the sequelae of underweight status among children has been well-defined in recent decades, the causes of childhood malnutrition are complex and influenced by many contextual factors ranging from political instability to limited accessibility of food.

Following the genocide against the Tutsi in 1994, Rwanda faced a massive loss of health professionals and a destruction of health infrastructure [3]. Over the past two decades since the genocide, health outcomes in Rwanda have improved drastically. Expanded access to health facilities, clinicians, and community health workers, as well as international funding channeled toward broader systems strengthening efforts have led to precipitous declines in premature mortality among children under-five years [4]. Rwanda seems now on track to meet each of the health-related United Nations Millennium Development Goals [3]. However, child malnutrition has persisted in Rwanda and serious investigation into the causes is warranted.

One strategy that has demonstrated potential to improve health outcomes is performance-based financing (PBF) [5, 6]. PBF or pay-for-performance (P4P) is a health system financing strategy that Rwanda has implemented in the last decade [7]. In early 2000, donor support, aimed at rebuilding the country’s physical infrastructure, had begun to decline and user fees were reintroduced, resulting in decreased health care utilization, poor coverage, and inadequate incentives for health care providers. PBF operates by transferring money condition upon taking measureable action or achieving predetermined performance targets [8]. In Rwanda, the program was initially introduced between 2002 and 2004 in pilot districts and scaled-up to the national level in 2006, made possible due to Rwanda’s commitment to good government, which has been identified as an essential component to the success of the PBF approach [9].

Among the known associations with childhood malnutrition, maternal HIV status is thought to play an important role in countries where HIV/AIDS is endemic. The perinatal nutritional status of HIV positive mothers has been shown to be associated with infant birth weight, and the weight and development of her child from birth to adolescence [10]. A few studies have demonstrated a high prevalence of malnutrition among children of HIV positive mothers [11–13]. However, a study conducted in the Democratic Republic of Congo (DRC) suggested that maternal immunological and socioeconomic conditions had no significant impact on the child’s malnutrition status. Rather, the child’s sero-status and not the mothers, was the strongest determinant of malnutrition [14]. Similar results to the study in the DRC have been demonstrated in Malawi and Uganda [15, 16].

Therefore, it is crucial that the Rwandan health sector identifies its own correlates of malnutrition, taking into account the findings of other sub-Saharan African countries. Our study aimed to assess the impact of the PBF approach and other factors, including maternal HIV status, on the prevalence of three classifications of malnutrition (stunting, wasting and underweight) in children under-5 years in Rwanda.

## Methods

### Study design

The study was conducted by the University of Rwanda, School of Public Health, in collaboration with The World Bank, the Rwanda Ministry of Health and Instituto Nacional de Salud Pública (INSP) of Mexico. The current assessment took advantage of a prospective, quasi-experimental design that evaluated the national expansion of the PBF program over 2006 and 2008. PBF built on existing contracts between government health authorities and health care providers and were scaled-up nationally in 2008. In 2005, districts that did not have a PBF scheme in their health facilities were identified and phased into the program. In brief, these districts were randomly assigned to two stages: phase I (districts which began receiving PBF in 2006) and phase II (districts which began receiving PBF in 2008). Phase 0 districts were those that had implemented PBF in the pilot phase prior to 2006. A detailed description of the methodology for the evaluation of PBF in Rwanda is documented elsewhere [5].

### Study participants

Data on status of malnutrition was taken from the interviewer-administered Rwanda General Health and HIV household survey that collected information on demographics, socio-economic characteristics and recorded children’s anthropometric measurements in 2006, when there were three phases of the PBF intervention: phase 0 (implemented PBF in 2002 pilot), phase I (implemented PBF in 2006) and phase II districts (implemented PBF in 2008). This household survey was administered to a sample of HIV positive patients and their households, and a smaller set of neighboring households. HIV/AIDS patients were identified either by contacting the health facility where they received care or via association of People Living With HIV/AIDS (PLWHA). They were randomly selected proportional to the number of HIV positive patients registered with health facilities delivering HIV prevention and treatment services. Informed consent was obtained from the patients before interviewing their household. Children were included in this analysis if measurements for height and weight were obtained and they resided in a district where PBF was implemented at the time (phase 0 or phase I) or not yet implemented (phase II).

### Data collection and variables

Nutritional status of participants was evaluated by means of anthropometric indices calculated on the basis of the child’s age, height and weight measurements. During home visits, height and weight of children were measured and recorded using a height gauge and a scale. The age was calculated in months starting from the date of birth to the day of participation in this study. The Z-scores for height-for-age (HAZ), weight-for-age (WAZ), weight-for-height (WHZ), and body mass index-for-age (BMI/A) were computed based on the World Health Organization (WHO) 2006 Child Growth Standard [17]. The interviews were conducted with a child’s mother or primary caregiver to obtain demographic and other health-related information.

The anthropometric indices above served as the outcomes for this analysis. We classified children nutritional status using a cutoff Z-score of below negative two (−2) standard deviations from the median of the reference population (Z-score < −2) according to the 2006 WHO Growth Reference Chart. Scores below −2 led to underweight, stunted or wasted children. Missing values were given for children with unknown dates of birth and for outlier z-scores: WAZ > 5 or WAZ < −6; HAZ > 6 or HAZ < −6; WHZ > 5 or WHZ < −5 SD. Explanatory variables were measured at child level (sex, age, health insurance, HIV status and recent illness), mother level (age, education, marital status, HIV status, and emotional social and physical functioning) and at the household level (parity, health expenditures and PBF).

### Data analysis

Summary statistics were used to describe the distributions of key variables within the sample. We estimated random intercept logistic regression models where each anthropometric measure (WAZ, HAZ and WHZ) was regressed against an indicator variable for whether the child’s mother was HIV infected or not, a health facility fixed effect, and a series of child, mother and household characteristics. Multiple logistic regression models with health facility fixed effect were used to evaluate the effect of mother’s HIV serological status on their children nutrition status. Model I is adjusted for child characteristics, model II is adjusted for child characteristics and model III is adjusted for child characteristics, maternal characteristics, household characteristics and whether the household resided in a PBF district (phase I or 0). No sensitivity analyses were performed and all data that met the inclusion criteria was included. All analyses were conducted in STATA version 11.0.

### Ethics

The study protocol was approved by both the Rwandan National AIDS Control Commission and the Rwandan Ethical Committee.

## Results

### Sample description

A total of 713 children between 0 and 60 months from 557 households were included in our sample (Table 1). Of these households, 89 (16%) were located in phase 0 districts, 233 (42%) in phase I, and 235 (42%) in phase II districts. Of the total sample, 11.4% were under-6 months of age and 8.7% were between 6 and 11 months. The percentages of male and female children were similar. In the sample, 5.1% of all children were HIV positive and mother’s reported that 31.3% of children were sick in the six months prior to the survey and/or were admitted to hospital within the past 12 months. Almost 80% of child participants had health insurance. The overall prevalence of wasting was 9.0%, of stunting was 57.9%, and of underweight status was 22.7%.

**Table 1:** **Demographic and clinical characteristics of 762 children under-5 years from 557 unique households**

Variable	Count (%)*
**Child characteristic**	
**Age**	
0-5 months	81 (11.4)
6-11 months	62 (8.7)
12-23 months	117 (16.4)
24-35 months	152 (21.3)
36-47 months	146 (20.5)
48-60 months	155 (21.7)
**Sex**	
Male	351 (49.2)
Female	362 (50.8)
**Health insurance**	
No	146 (20.7)
Yes	558 (79.3)
**HIV status**	
Negative	676 (94.9)
Positive	36 (5.1)
**Recent illness**	
No	489 (68.7)
Yes	223 (31.3)
**Performance-based financing**	
Lives in Phase II district	235 (42.0)
Lives in Phase 0 district	89 (16.0)
Lives in Phase I district	233 (42.0)
**Outcome measure**	
**Weight-for-age (Underweight)**	
<−2 SD	157 (22.7)
≥ − 2 SD	535 (77.3)
**Height-for-age (Stunting)**	
<−2 SD	356 (57.9)
≥ − 2 SD	259 (42.1)
**Weight-for-height (Wasting)**	
<−2 SD	56 (9.0)
≥ − 2 SD	569 (91.0)

*Totals do not necessarily add to total sample size given that missing values were generated if Z-scores were grater than 5 in magnitude.

SD: Standard deviation.

### Associations with underweight, wasting and stunting

Results of the logistic regression models are presented in Tables 2, 3 and 4 to model the outcomes of underweight, stunting and wasting, respectively. Three models (I, II, III) are presented for each of the malnutrition outcomes. Of the variables tested, there were no significant associations with underweight children under-5 years (Table 2). Table 3 shows that children of 12–23 months were significantly more likely than children 0–5 months to be stunted (Adjusted Odds Ratio [AOR]: 2.83; 95% Confidence Interval [CI]: 1.33-5.92, in model III). Finally, children 12–23 months (AOR: 0.28; 95% CI: 0.08-0.96) and 48–60 months (AOR: 0.17; 95% CI: 0.04-0.64) were significantly less likely to be wasted compared to children 0–5 months.

**Table 2:** **Three models of multivariable logistic regression investigating factors associated with underweight children under-5 years of age in Rwanda**

	I: AOR (95% CI)	II: AOR (95% CI)	III: AOR (95% CI)
**Child characteristics**			
**Sex**			
Male	1.00 (−−)	1.00 (−−)	1.00 (−−)
Female	1.13 (0.79 – 1.62)	1.00 (0.68 - 1.48)	1.06 (0.71 - 1.58)
**Age in months**			
0-5 months	1.00 (−−)	1.00 (−−)	1.00 (−−)
6-11 months	0.55 (0.24 - 1.28)	0.63 (0.26 - 1.55)	0.58 (0.23 - 1.46)
12-23 months	0.57 (0.28 - 1.18)	0.58 (0.26 - 1.28)	0.58 (0.26 - 1.30)
24-35 months	0.76 (0.38 - 1.51)	0.86 (0.41 - 1.79)	0.83 (0.39 - 1.76)
36-47 months	0.68 (0.34 - 1.38)	0.62 (0.29 - 1.33)	0.60 (0.27 - 1.32)
48-60 months	0.59 (0.30 - 1.16)	0.52 (0.24 - 1.15)	0.54 (0.24 - 1.19)
**Health insurance**			
No	1.00 (−−)	1.00 (−−)	1.00 (−−)
Yes	0.96 (0.60 - 1.54)	1.05 (0.63 - 1.76)	1.09 (0.64 - 1.86)
**HIV status**			
Negative	1.00 (−−)	1.00 (−−)	1.00 (−−)
Positive	1.17 (0.51 - 2.66)	1.38 (0.56 - 3.38)	1.30 (0.50 - 3.41)
**Recent illness**			
No	1.00 (−−)	1.00 (−−)	1.00 (−−)
Yes	1.01 (0.67 - 1.53)	0.97 (0.63 - 1.50)	1.02 (0.65 - 1.59)
**Maternal characteristics**			
**Education**			
None		1.00 (−−)	1.00 (−−)
Primary		1.23 (0.76 - 2.00)	1.31 (0.80 - 2.16)
> Primary		0.90 (0.42 - 1.95)	0.84 (0.38 - 1.88)
**Age (continuous)**		1.00 (0.97 - 1.04)	1.00 (0.97 - 1.04)
**Marital status**			
Married		1.00 (−−)	1.00 (−−)
Divorced/widowed		1.18 (0.71 - 1.95)	1.23 (0.73 - 2.06)
Never married		0.74 (0.35 - 1.57)	0.79 (0.37 - 1.70)
**HIV status**			
Negative		1.00 (−−)	1.00 (−−)
Positive		0.72 (0.46 - 1.12)	0.77 (0.49 - 1.22)
**Emotional problems**			
No		1.00 (−−)	1.00 (−−)
Yes		1.00 (1.00 - 1.00)	1.00 (1.00 - 1.00)
**Social functioning**			
High		1.00 (−−)	1.00 (−−)
Low		1.00 (0.99 - 1.01)	1.00 (0.99 - 1.01)
**Physical health**			
Good		1.00 (−−)	1.00 (−−)
Poor		1.00 (1.00 - 1.00)	1.00 (1.00 - 1.00)
**Household characteristics**			
**Catastrophic health expenditure**			
<20% income spent			1.00 (−−)
20% - 40% income spent			0.75 (0.31 - 1.84)
≥ 40% income spent			1.42 (0.61 - 3.32)
**Have other children under 5 years**			
No			1.00 (−−)
Yes			1.16 (0.89 - 1.51)
**Performance-based financing**			
Lives in Phase II district			1.00 (−−)
Lives in Phase 0 district			0.87 (0.48 - 1.59)
Lives in Phase I district			0.88 (0.57 - 1.35)

AOR: Adjusted Odds Ratio; CI: Confidence Interval.

I: Adjusted for child characteristics.

II: Adjusted for child characteristics and maternal characteristics.

III: Adjusted for child characteristics, maternal characteristics, household characteristics and PBF.

**Table 3:** **Three models of multivariable logistic regression investigating factors associated with stunting among children under-5 years of age in Rwanda**

	I: AOR (95% CI)	II: AOR (95% CI)	III: AOR (95% CI)
**Child characteristics**			
**Sex**			
Male	1.00 (−−)	1.00 (−−)	1.00 (−−)
Female	0.90 (0.65 - 1.26)	1.03 (0.73 - 1.46)	1.01 (0.71 - 1.44)
**Age in months**			
0-5 months	1.00 (−−)	1.00 (−−)	1.00 (−−)
6-11 months	1.35 (0.62 – 2.92)	1.28 (0.57 - 2.87)	1.25 (0.56 - 2.81)
12-23 months	**2.70 (1.33 - 5.49)**	**2.78 (1.31 - 5.92)**	**2.83 (1.33 - 5.92)**
24-35 months	1.04 (0.54 - 2.01)	1.06 (0.53 - 2.12)	1.06 (0.53 - 2.12)
36-47 months	1.60 (0.83 - 3.08)	1.40 (0.70 - 2.81)	1.39 (0.69 - 2.80)
48-60 months	1.49 (0.79 – 2.83)	1.68 (0.84 - 3.38)	1.71 (0.85 - 3.45)
**Health insurance**			
No	1.00 (−−)	1.00 (−−)	1.00 (−−)
Yes	0.83 (0.53 - 1.30)	1.01 (0.63 - 1.62)	1.02 (0.63 - 1.65)
**HIV status**			
Negative	1.00 (−−)	1.00 (−−)	1.00 (−−)
Positive	1.82 (0.76 - 4.36)	1.55 (0.59 - 4.05)	1.59 (0.60 - 4.18)
**Recent illness**			
No	1.00 (−−)	1.00 (−−)	1.00 (−−)
Yes	1.10 (0.75 - 1.51)	1.03 (0.70 - 1.52)	1.04 (0.70 - 1.55)
**Maternal characteristics**			
**Education**			
None		1.00 (−−)	1.00 (−−)
Primary		1.21 (0.77 - 1.89)	1.27 (0.81 - 1.99)
> Primary		1.09 (0.56 - 2.13)	1.12 (0.57 - 2.18)
**Age (continuous)**		0.99 (0.96 - 1.03)	0.99 (0.96 - 1.03)
**Marital status**			
Married		1.00 (−−)	1.00 (−−)
Divorced/widowed		1.08 (0.69 - 1.70)	1.10 (0.69 - 1.74)
Never married		1.04 (0.54 - 2.00)	1.00 (0.51 - 1.96)
**HIV status**			
Negative		1.00 (−−)	1.00 (−−)
Positive		1.07 (0.72 - 1.59)	1.08 (0.72 - 1.62)
**Emotional problems**			
No		1.00 (−−)	1.00 (−−)
Yes		1.00 (1.00 - 1.01)	1.00 (1.00 - 1.01)
**Social functioning**			
High		1.00 (−−)	1.00 (−−)
Low		1.00 (0.99 - 1.01)	1.00 (0.99 - 1.01)
**Physical health**			
Good		1.00 (−−)	1.00 (−−)
Poor		1.00 (1.00 - 1.00)	1.00 (1.00 - 1.00)
**Household characteristics**			
**Catastrophic health expenditure**			
<20% income spent			1.00 (−−)
20% - 40% income spent			0.93 (0.45 - 1.91)
≥ 40% income spent			0.82 (0.34 - 1.95)
**Have other children under 5 years**			
No			1.00 (−−)
Yes			1.10 (0.86 - 1.4)
**Performance-based financing**			
Lives in Phase II district			1.00 (−−)
Lives in Phase 0 district			0.90 (0.55 - 1.48)
Lives in Phase I district			0.98 (0.67 - 1.44)

AOR: Adjusted Odds Ratio; CI: Confidence Interval.

I: Adjusted for child characteristics.

II: Adjusted for child characteristics and maternal characteristics.

III: Adjusted for child characteristics, maternal characteristics, household characteristics and PBF.

Bold typeface indicates factors significantly associated with malnutrition at the 0.05 significance level.

**Table 4:** **Three models of multivariable logistic regression investigating factors associated with wasting among children under-5 years of age in Rwanda**

	I: AOR (95% CI)	II: AOR (95% CI)	III: AOR (95% CI)
**Child characteristics**			
**Sex**			
Male	1.00 (−−)	1.00 (−−)	1.00 (−−)
Female	0.94 (0.53 - 1.65)	0.68 (0.36 - 1.30)	0.64 (0.33 - 1.23)
**Age in months**			
0-5 months	1.00 (−−)	1.00 (−−)	1.00 (−−)
6-11 months	0.44 (0.13 - 1.53)	0.50 (0.14 - 1.85)	0.42 (0.11 - 1.60)
12-23 months	**0.26 (0.08 – 0.80)**	**0.28 (0.08 - 0.96)**	**0.28 (0.08 - 0.96)**
24-35 months	0.53 (0.20 - 1.43)	0.51 (0.17 - 1.50)	0.45 (0.15 - 1.36)
36-47 months	0.42 (0.15 - 1.15)	0.43 (0.14 - 1.32)	0.38 (0.12 - 1.20)
48-60 months	**0.35 (0.13 - 0.95)**	**0.20 (0.06 - 0.71)**	**0.17 (0.04 - 0.64)**
**Health insurance**			
No	1.00 (−−)	1.00 (−−)	1.00 (−−)
Yes	0.95 (0.46 - 1.98)	0.84 (0.37 - 1.89)	0.85 (0.37 - 1.93)
**HIV status**			
Negative	1.00 (−−)	1.00 (−−)	1.00 (−−)
Positive	0.89 (0.25 - 3.19)	0.80 (0.16 - 3.89)	1.00 (0.20 - 5.03)
**Recent illness**			
No	1.00 (−−)	1.00 (−−)	1.00 (−−)
Yes	1.46 (0.80 - 2.68)	1.29 (0.65 - 2.56)	1.21 (0.60 - 2.44)
**Maternal characteristics**			
**Education**			
None		1.00 (−−)	1.00 (−−)
Primary		0.68 (0.32 - 1.43)	0.73 (0.34 - 1.56)
> Primary		0.62 (0.18 - 2.13)	0.64 (0.18 - 2.22)
**Age (continuous)**		0.98 (0.93 - 1.03)	0.98 (0.92 - 1.04)
**Marital status**			
Married		1.00 (−−)	1.00 (−−)
Divorced/widowed		1.26 (0.59 - 2.38)	1.24 (0.56 - 2.72)
Never married		0.93 (0.29 - 2.96)	0.77 (0.23 - 2.54)
**HIV status**			
Negative		1.00 (−−)	1.00 (−−)
Positive		1.37 (0.64 – 2.93)	1.28 (0.59 - 2.77)
**Emotional problems**			
No		1.00 (−−)	1.00 (−−)
Yes		0.99 (0.98 - 1.00)	0.99 (0.98 - 1.00)
**Social functioning**			
High		1.00 (−−)	1.00 (−−)
Low		0.99 (0.98 - 1.00)	0.99 (0.98 - 1.00)
**Physical health**			
Good		1.00 (−−)	1.00 (−−)
Poor		1.00 (1.00 - 1.00)	1.00 (1.00 - 1.00)
**Household characteristics**			
**Catastrophic health expenditure**			
<20% income spent			1.00 (−−)
20% - 40% income spent			1.08 (0.27 – 4.32)
≥ 40% income spent			1.77 (0.44 – 7.04)
**Have other children under 5 years**			
No			1.00 (−−)
Yes			0.98 (0.68 – 3.23)
**Performance-based financing**			
Lives in Phase II district			1.00 (−−)
Lives in Phase 0 district			1.48 (0.68 - 3.23)
Lives in Phase I district			**0.43 (0.19 - 0.97)**

AOR: Adjusted Odds Ratio; CI: Confidence Interval.

I: Adjusted for child characteristics.

II: Adjusted for child characteristics and maternal characteristics.

III: Adjusted for child characteristics, maternal characteristics, household characteristics and PBF.

Bold typeface indicates factors significantly associated with malnutrition at the 0.05 significance level.

With respect to the main objective of the study, children residing in a PBF implemented district were significantly less likely to be stunted (AOR: 0.43; 95% CI: 0.19-0.97) compared to districts that had not yet received the PBF intervention. Neither of the other two forms of malnutrition in children under-5 years (underweight and stunting) were found to be significantly more or less likely to be prevalent in a district with PBF. Lastly, our results show that maternal HIV infection had no significant effect on any forms of childhood malnutrition measured in our study.

## Discussion

This study investigated the impact of PBF and other factors associated with three forms of malnutrition (underweight, stunting and wasting) among children under-5 years in Rwanda. We specifically hypothesized a protective association between malnutrition and the implementation of PBF after controlling for maternal HIV status. Our study found that children born to HIV-infected mothers were no more likely to be malnourished than children born to HIV-negative mothers and that children residing in districts that had implemented the PBF scheme were significantly less likely to meet the criteria for wasting.

These findings are generally supported by existing literature on PBF. PBF aims to increase access to care, and increase patient utilization of preventive care services, thus improving health service delivery. Similarly, Basinga and colleagues found that PBF in Rwanda improved both the use of maternal and child health services [5]. The authors’ model showed that facilities receiving PBF had a 23% increase in the number of institutional deliveries and increases in the number of preventive care visits by children aged 23 months or younger and between 24 months and 59 months. Although we only observed a relationship between PBF and one form of malnutrition, the significantly protective association with wasting can most likely be attributed to an increased number of visits and engagement with the health system in districts with PBF. Malnutrition is not often a condition in which mothers often seek care for their children and many forms of the condition remain under-diagnosed [2]. However, we hypothesize that the implementation of PBF has led to an increase in preventative service visits in which a health care provider may recognize wasting children and intervene appropriately. In contrast to stunting and underweight, which are typically treated as more chronic conditions, wasting is generally considered a more acute form of malnutrition. An increase in preventative service visits, as speculated under the PBF scheme may allow for the treatment of wasting by a health care professional, while underweight and stunting take longer to address the chronic underlying causes of disease.

Our finding of associations between wasting and stunting and age of the child is well supported in the literature. Infants often do not receive sufficient nutrients in the breastfeeding weaning period, which often consists of foods with substandard levels of macro- and micronutrients [18]. Weaning foods high in starch and low in energy density affect the child’s likelihood of being marasmic – a condition also in positive correlation with stunting. Studies in the East Africa region have found that older children have a higher prevalence of stunting than children who are younger even after controlling for urban and rural differences in feeding practice [19]. It is clear that the onset of stunting may occur early on, yet may not be detected until the infant is at least 12 months old [20], which aligns with our findings that children between 12–24 months old were more likely to be stunted than children who were under 6 months old. Other studies have had similar conclusions regarding the mental and socioeconomic wellbeing of the mother and the nutritional status of her child [21, 22].

One limitation of this study is that there were a number of confounding factors during the time of PBF scale-up, including increased health insurance coverage (Mutuelles de Santé) and overall improvements in the healthcare system [7]. However, when access to health insurance was controlled for, our study found no association between health insurance access and childhood malnutrition. A review of the literature on performance-based payments for the purpose of improving health service delivery found similarly mixed results. These authors noted that more research needs to be done using case studies or comparative studies between facilities where PBF is implemented, and where it is not [23]. In our study in Rwanda, persons in all districts have access to health insurance, therefore differences between different districts might correlate with presence or absence of PBF (among other factors), but would not correlate with access to health insurance. Our study also observed some marked differences in the prevalence HIV with that which is known for the population of Rwanda. First, 5.1% of the children were HIV positive while the Center for Treatment and Research on AIDS, Malaria, Tuberculosis and Other Epidemics (TRAC Plus) reports that in 2008, 7.0% of infants born to HIV-infected mothers tested positive for HIV at 18 months [24]. Given that HIV status was self-reported by mothers rather than participant tested, this could account for the difference and is a recognized limitation to our study. Finally, our study observed much more stunting (58.42%) than results found in the RDHS 2005 (45% below −2 standard deviations) [25]. Nonetheless, as this was a study to better understand associations between variables, having oversampled stunted children should have minimal impact on the validity of the study.

## Conclusions

In summary, our study has shown a protective association between implementing PBF, an innovative health financing scheme, and wasting among children in Rwanda. We did not, as expected, reveal a relationship between maternal sero-positivity and child malnutrition. Child malnutrition remains a critical problem in Rwanda and further studies are needed to explore additional associated factors and evaluate the effectiveness of interventions, such as PBF, on reducing the burden of childhood malnutrition. We add to the literature a call to support better mental, emotional, and social wellbeing assessments at every clinical visit; and advocate that health care workers be better equipped with training in psychosocial assessment and support. With respect to PBF, it is clear that more research is needed in order to make more detailed recommendations on the impact of PBF on specific health outcomes. We recommend a more thorough evaluation of the impact of implementing PBF in Rwanda on malnutrition and other preventive health services.
